# Evaluation of Potential Average Daily Doses (ADDs) of PM_2.5_ for Homemakers Conducting Pan-Frying Inside Ordinary Homes under Four Ventilation Conditions

**DOI:** 10.3390/ijerph14010078

**Published:** 2017-01-13

**Authors:** Seonyeop Lee, Sol Yu, Sungroul Kim

**Affiliations:** Department of Environmental Health Sciences, Soonchunhyang University, Asan 31538, Korea; phantom3586@gmail.com (S.L.); solsol0914@gmail.com (S.Y.)

**Keywords:** PM_2.5_, average daily doses, indoor, ventilation, pan-frying

## Abstract

Several studies reported that commercial barbecue restaurants likely contribute to the indoor emission of particulate matters with a diameter of 2.5 micrometers or less (PM_2.5_) while pan-frying meat. However, there is inadequate knowledge of exposure level to indoor PM_2.5_ in homes and the contribution of a typical indoor pan-frying event. We measured the indoor PM_2.5_ concentration and, using Monte-Carlo simulation, estimated potential average daily dose (ADD) of PM_2.5_ for homemakers pan-frying a piece of pork inside ordinary homes. Convenience-based sampling at 13 homes was conducted over four consecutive days in June 2013 (*n* = 52). Although we pan-fried 100 g pork for only 9 min, the median (interquartile range, IQR) value was 4.5 (2.2–5.6) mg/m^3^ for no ventilation and 0.5 (0.1–1.3) mg/m^3^ with an active stove hood ventilation system over a 2 h sampling interval. The probabilities that the ADDs from inhalation of indoor PM_2.5_ would be higher than the ADD from inhalation of PM_2.5_ on an outdoor roadside (4.6 μg/kg·day) were 99.44%, 97.51%, 93.64%, and 67.23%, depending on the ventilation conditions: (1) no window open; (2) one window open in the kitchen; (3) two windows open, one each in the kitchen and living room; and (4) operating a forced-air stove hood, respectively.

## 1. Introduction

According to statistics reported in 2013, the number of deaths in South Korea due to cancer was 149 per 100,000: 34 from lung cancer, 22.6 from liver cancer, 18.2 from stomach cancer, and 74.2 from other cancers [1]. Yu et al. reported in 2006 that exposure to indoor toxic compounds emitted during cooking activity at home was likely a risk factor increasing the incidence of lung cancer among nonsmoking women [2]. Particulate matters with a diameter of 2.5 micrometers or less PM_2.5_ particles emitted during pan-frying can be harmful to human health due to their relatively small size and corresponding ability to penetrate deep into the lungs and enter the blood stream unfiltered. The results of many studies have indicated that PM_2.5_, particulate matter with an aerodynamic diameter equal to or smaller than 2.5 μm, could be produced during cooking activities [3,4,5,6], and it has been reported that acute reduction of lung function was associated with exposure to PM_2.5_ during cooking activities [7,8]. In 2010, the International Agency for Research on Cancer (IARC), a specialized cancer agency of the World Health Organization (WHO), reported that emissions from high-temperature frying are probably carcinogenic to humans (Group 2A) although there is limited evidence in humans for the carcinogenicity of emissions from high-temperature frying. However, there is sufficient evidence in experimental animals for the carcinogenicity of emissions from high-temperature unrefined rapeseed oil [9]. Therefore, cooking related indoor PM_2.5_ levels should be carefully monitored due to their potential harmful characteristics, as we described above.

According to a report from the Ministry of Environment of South Korea [10], the amount of PM_2.5_ produced by pan-frying meats was 3022 tons/year, assuming a meat consumption rate of 31.3 kg/year per person. Because pan-fried pork belly is a popular dish among Koreans at home [11], it is probable that homemakers are exposed to high levels of PM_2.5_ emitted during the pork pan-frying process. They are also potentially exposed to secondary particles formed by combination of chemicals derived from the oxidation of primary gases produced during pan-frying.

Many researchers have reported that commercial barbecue restaurants likely contribute to the emission of toxic chemicals and PM_2.5_ into indoor and outdoor air during the commercial food pan-frying or pan-frying process [12]. According to a recent report in South Korea, PM_2.5_ emitted from commercial restaurants serving barbecue accounted for 8.7% of all the PM_2.5_ produced in Gyeonggi Province [13]. However, information on the indoor PM_2.5_ concentration in homes contributed to by residential pan-frying, and the degree of PM_2.5_ reduction related to the ventilation conditions prevalent in homes, is insufficient.

We conducted this study to evaluate the potential indoor exposure and average daily dose of homemakers to PM_2.5_ generated during pan-frying meat in ordinary South Korean homes. To this end, four different arrangements of ventilation were simulated.

## 2. Materials and Methods

### 2.1. Sampling Sites

Convenience-based sampling in 13 homes (six single houses, six apartments, and one multiunit) was conducted over four consecutive days to measure the indoor PM_2.5_ concentration levels. Our experiments (*n* = 52) were conducted from June to December 2013 in Cheonan and Seoul, South Korea.

The total floor area of these homes ranged from 52.8 to 112.2 m^2^ and the approximate height from floor to ceiling was 2.5 m or less in each home. We conducted four measurements per house per day. During our experiments, operating a fan or air conditioning system was not allowed. For one day before the experiment in each home, pan-frying other meats or fishes was not allowed by our monitoring agents. Characteristics of our sampling sites are summarized in Table 1.

### 2.2. Pan-Frying Process and Ventilation Conditions Applied

Our experiments were done by simulating the barbequing of pork belly (100 g) for 9 min over a 2-h measurement period per trial under four different ventilation conditions. With our pre-established standard operating protocol, a portion of pork belly (100 g) was pan-fried for 9 min: 3 min on Side A, 3 min on Side B; then 1.5 min for Side A again, and a final 1.5 min for Side B again. We used the same nonstick pans for every experiment without cooking oil. Pan-frying in all houses was done with their gas-ranges using natural gas (41.0–44.4 MJ/Nm^3^) supplied by our national distributor, Korea Gas Corporation [14].

**Figure 1:**
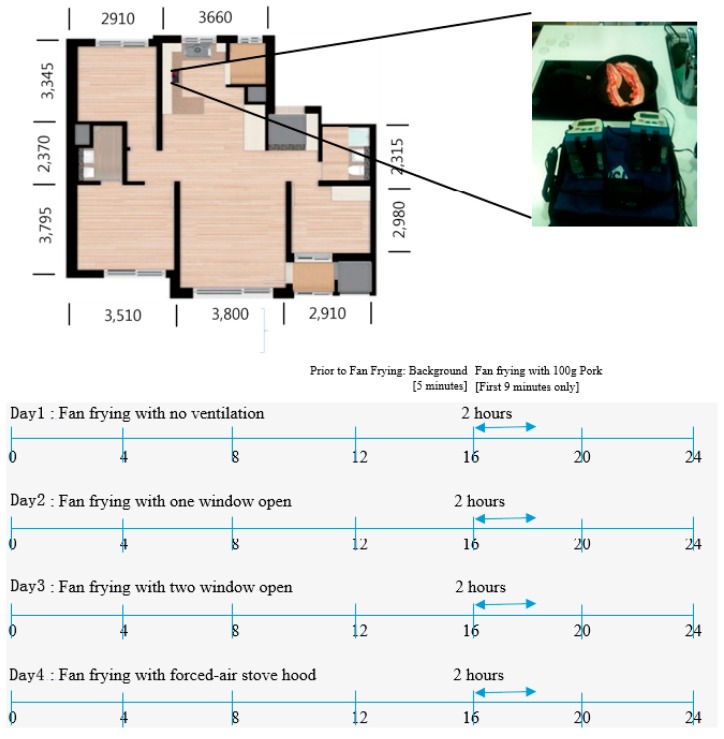
Floor layout of a typical sampling site (unit of length: mm) and schematic of sampling frequency and duration.

The ventilation conditions were as follows: (1) No windows open; (2) one window, of size 0.5 ± 0.28 m^2^, open in the kitchen was selected as the simplest natural ventilation method, where no forced-air stove-hood operation system is available; (3) two windows open, both in the kitchen and the living room (window size on the opposite side of the kitchen, 2.3 ± 0.20 m^2^), likely increasing natural ventilation by allowing air circulation and expelling it from both ends (kitchen and living room); and (4) forced-air stove-hood operating during the entire pan-frying process. All households had a gas-range hood and windows, and no other windows were opened during the experiment. Floor area (5.3–11.2 m^2^) of the kitchen was approximately 10% of entire floor area (52.8–112 m^2^) and no separation door existed between the kitchen and living room of any of the houses (Figure 1).

To avoid the carry-over effect of PM_2.5_ concentrations between simulations and to minimize the effect of non-target sources contributing to our PM_2.5_ measurement results [15], we took measurements of the background PM_2.5_ concentrations inside the kitchen and outside the kitchen window for five minutes before and after conducting our experiments. Later, we subtracted the indoor background concentration from our indoor values.

### 2.3. PM_2.5_ Measurement

We used real-time PM_2.5_ monitors (Sidepak, TSI, Shoreview, MN, USA) to measure the indoor PM_2.5_ levels (flow rate 1.7 L/min) as a stationary sampler. Every day prior to PM_2.5_ monitoring, we performed zero calibration and checked the flow rate [16]. Our monitor was 50 cm from the stove hood fan and 1 m above the kitchen floor. We used two Sidepaks for each experiment. We applied 0.65 as a Sidepak correction factor of PM_2.5_ concentrations over the pork pan-frying process according to the previous study results, reporting real-time particle monitor calibration factors for multiple indoor emission sources by comparing the outcome of real-time laser photometers, including Sidepak, and a filter-based PM_2.5_ gravimetric sampler to quantify the monitor calibration factors (CFs) [17]. We kept a minimum distance of 50 cm between the two Sidepaks. The final distributions of PM_2.5_ concentrations, according to four ventilation conditions, were obtained from the 13 PM_2.5_ median concentration values for 13 sampling sites. At each pan-frying trail, we took a PM_2.5_ concentration value every 1 min over a 2 h sampling period.

### 2.4. Average Daily Dose

The PM_2.5_ doses inhaled by housewives on monitoring days, under four different ventilation conditions, was determined using Equation (1), adopted from the average daily dose calculation handbook [18] of U.S. Environmental Protection Agency (EPA), as well as from the Korean exposure handbook [19].

(1) $$\mathrm{ADD}\;(\mathrm{mg}/\mathrm{kg}\cdot \mathrm{day})=\frac{C\times \mathrm{IR}\times \mathrm{ET}}{\mathrm{BW}\times \mathrm{AT}\times 1000}$$

C: Arithmetic mean concentration of the PM_2.5_ (mg/m^3^); IR: inhalation rate (L/min); BW: body weight (kg); ET: exposure time (min); AT: average time (days).

ADD is the average daily dose (milligrams per kilogram per day, mg/kg·day) by inhalation. Here, C is the average value of the 13 median PM_2.5_ concentrations from pan-frying pork (mg/m^3^) measured over a 2 h period in houses. IR is the estimated air inhalation rate (L/min) and BW, ET, and AT are the estimated body weight (kg), exposure time (min), and average time (days) for housewives, respectively [20]. The PM_2.5_ ADD by inhalation obtained for housewives on monitoring days, with different ventilation conditions, was compared with their estimated PM_2.5_ ADD by inhalation of roadside PM_2.5_ levels.

According to the Korean exposure handbook [19], we applied the inhalation rate 10.9 ± 3.8 L/min for adult women (average body weight 56.4 ± 7.81 kg) assuming pan-frying pork at home once a week for 35 years (until their retirement at the age of 60 years). We also assumed that the pork pan-frying related cooking time was 65 min [21]. For the comparison purposes, we used the 24-h outdoor PM_2.5_ standard (0.05 mg/m^3^) from the Korea National Ambient Air Quality scale [22]. For this comparison, we assumed that the women worked outside and they were exposed to roadside PM_2.5_ for 8 h/day (daytime) and 40 h/week for 35 years (until their retirement at the age of 60 years). For calculation of ADD by inhalation of roadside PM_2.5_, we applied the same inhalation rate (10.9 ± 3.8 L/min) for women working outside, near roadsides [19]. Under assumptions of 8 h working time outside for 7 days over 35 years, we obtained 4.6 mg/kg·day.

### 2.5. Probabilistic Modeling: ADD Distribution by Monte-Carlo Simulation

Using a Monte-Carlo simulation with Crystal Ball (version 11, Oracle, Redwood Shores, CA, USA), we compared probabilistic distributions of ADDs for inhalation of indoor as well as outdoor PM_2.5_ particles for homemakers. For this simulation, we assumed that indoor or outdoor PM_2.5_ concentrations were log-normally distributed while the distributions of body weight and inhalation rate were normal and that of exposure duration was a constant value. To obtain the probabilistic distribution, we repeated the simulation procedure 10,000 times.

### 2.6. Statistical Analysis

The distributions of indoor PM_2.5_ concentrations under each different ventilation condition was compared with the results obtained with no ventilation using the Wilcoxon rank-sum test.

## 3. Results

### 3.1. Indoor PM_2.5_ Levels According to Ventilation Conditions

We obtained different median (interquartile range, IQR) PM_2.5_ concentrations (*n* = 13 per each ventilation scenario), over a 2 h sampling period, in relation to the different ventilation conditions: 4.5 (2.2–5.6) mg/m^3^ for no ventilation, 1.8 (1.4–3.3) mg/m^3^ or 1.9 (0.4–2.5) mg/m^3^ for one or two windows open, and 0.5 (0.1–1.3) mg/m^3^ with the forced-air stove hood ventilator operating (Table 2). In detail, the median (IQR) concentrations of indoor PM_2.5_ during the first 9 min fan-prying period were 5.1 (3.0–9.2), 5.0 (1.7–7.0), 3.8 (1.3–6.2), and 1.16 (0.2–2.3) mg/m^3^, respectively. The corresponding dissipation kinetics after cooking was completed were 38.2 (26.6–79.4), 47.4 (17.0–84.6), 54.7 (20.1–99.9) and 55.2 (6.5–78.7) μg/m^3^ (Table 3).

**Figure 2:**
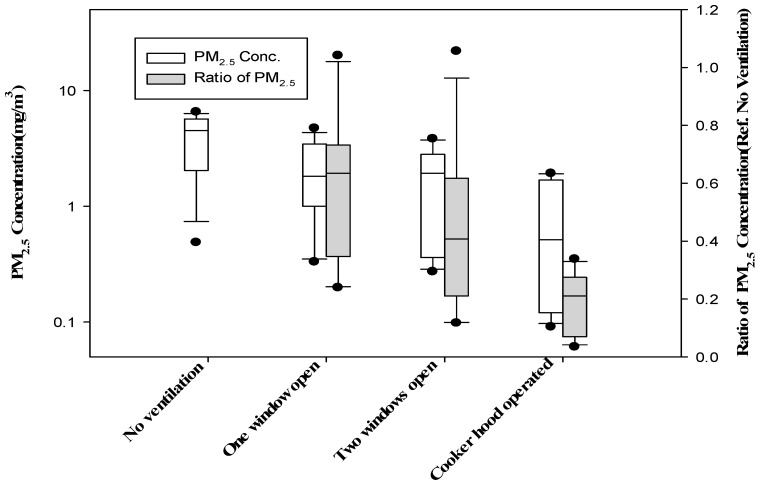
Distributions of median indoor particulate matters with a diameter of 2.5 micrometers or less (PM_2.5_) concentrations obtained at each sampling site according to ventilation condition and distribution of ratios of PM_2.5_ concentrations obtained with ventilation, to those without ventilation; Concentrations were lower than the reference (*p* < 0.05).

The median (interquartile range) of the ratio of PM_2.5_ concentrations with one or two windows open, or with cooker stove hood operating; to the concentrations without ventilation, for each paired observation for each home were 0.63 (0.40–0.69), 0.41 (0.23–0.56), or 0.17 (0.08–0.25), respectively (Figure 2).

### 3.2. Average Daily Dose (ADD) of Homemakers

**Figure 3:**
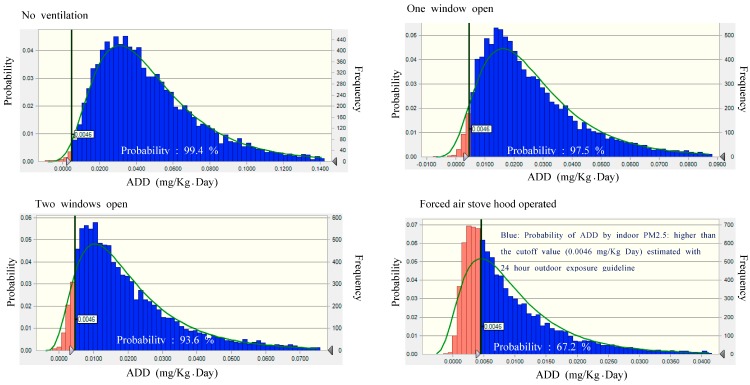
Distribution of average daily dose obtained from Monte Carlo simulation with the distribution of indoor PM_2.5_ levels observed under different ventilation conditions, and the probability of ADD by indoor PM_2.5_ which was higher (Blue) than the ADD (0.0046 mg/kg·day) estimate for roadside PM_2.5_.

On the basis of the arithmetic mean values of the PM_2.5_ concentrations observed during pork pan-frying, under the ventilation conditions and exposure scenario mentioned above, we obtained average daily PM_2.5_ doses of 48.1, 27.4, 21.2 and 10.0 μg/kg·day, respectively, while the dose from roadside PM_2.5_ was 4.6 μg/kg·day. Also, the median (IQR) value of ADD by inhalation of indoor PM_2.5_ due to exposure to the pan-frying process, from our Monte-Carlo simulation, was 41.7 (26.9–62.8), 22.1 (13.8–35.3), 16.4 (9.6–27.6) and 7.0 (3.8–12.7), according to the corresponding ventilation condition, separately (Table 2). The probabilities that the ADDs from inhalation of indoor PM_2.5_ would be higher than the ADD by inhalation of outdoor roadside PM_2.5_ (4.6 μg/kg·day) were 99.4%, 97.5%, 93.6% and 67.2%, depending on the ventilation conditions (no ventilation, one window open, two windows open, stove hood operating, respectively) (Figure 3).

## 4. Discussion

Our study indicated that the levels of indoor PM_2.5_ due to pan-frying in home kitchens were significantly high. Even though we pan-fried 100 g pork for only 9 min, the median values of PM_2.5_ levels were 4.5 mg/m^3^ for no ventilation and 0.5 mg/m^3^ with operation of the forced-air stove hood ventilation system with a 2 h interval were approximately 10 to 90 times higher than the 24-h outdoor PM_2.5_ standard (0.05 mg/m^3^) from the Korea National Ambient Air Quality recommendations [22]. We used the Korean NAAQS as a reference for comparison because we do not have specific standards for indoor PM_2.5_ levels. Our findings indicate that the pork pan-frying process contributes substantially to indoor PM_2.5_ concentration levels at the ordinary Korean house and that this exposure is particularly elevated when ventilation is not available.

Our study results are supported by those of previous studies characterizing indoor PM exposure levels at Korean style barbeque restaurant. According to Lee et al. (2001) [23], the average levels of PM_2.5_ at the Korean barbecue style restaurant in Hong Kong were as high as 1.17 mg/m^3^, respectively. The level obtained in Hong Kong was similar to our study results (1.8 mg/m^3^: one window open, 1.9 mg/m^3^: two windows open, 0.5 mg/m^3^: forced air stove hood applied). Another Chinese study reported that personal exposure level to PM_2.5_ from burning biomass ranged from 0.136 to 0.162 mg/m^3^ [24].

Our median value (0.5 mg/m^3^) of PM_2.5_ concentrations, even with the best ventilation (i.e., operating a stove hood), was approximately 2–3 times higher than the value obtained from the Chinese study above [24], or the value (0.15 mg/m^3^) obtained from a casino [8], similar to the level obtained from the smoking areas (0.1 to 0.98 mg/m^3^) in computer game rooms or night clubs [25]. Because our PM_2.5_ results were obtained from sampling of stationary bases, rather than from personal monitoring, further exploration of the basis for the differences in distributions of PM_2.5_ levels between our study and their studies is limited. Nevertheless, our study revealed the potential for high levels of exposure to PM_2.5_ concentrations during pan-frying meat in ordinary households, especially in unventilated kitchens.

This study has some limitations. First, the sample size of our study was not large and we recruited the study homes at two cities, Seoul and Cheonan. Since Seoul and Cheonan are both highly urbanized areas, we assumed that the life patterns of people in the two cities were not different and there was no systemic difference in terms of cooking methods. The outcomes of the Monte Carlo simulation, which can provide the estimation of the probabilistic distribution of the ADDs of PM_2.5_ for the young, female Korean population, should be interpreted with care since we estimated the ADDs according to the Korean exposure handbook [19] and we applied the inhalation rate 10.9 ± 3.8 L/min for female, adult Korean women (average body weight 56.4 ± 7.81 kg) assuming that they pan-fry pork at home once a week for 35 years (until their retirement at the age of 60 years). Because we randomly selected 13 homes (four measurements per home) of typical house types (i.e., one multi-unit house, six single houses and six apartments) for young adult couples found in South Korea, the distributions of the concentrations should not be systematically biased. According to statistics from Korea [26], it has been reported that 47% of Koreans live in apartments while the rest of the population live in single or multi-unit houses. In our study, 46% of results were conducted in apartments (24 results from apartments, 24 results from single-units, and 4 results from a multi-unit house). Nevertheless, generalization of our study outcomes to other study populations may be limited. Second, because we conducted stationary monitoring in kitchens over a 2-h interval to determine a daily peak level, rather than 24-h personal sampling, we could not provide personal exposure levels. Third, we could not measure air exchange or ventilation rate because of limitations in our time and funding. The concentrations observed in the first 4 sites and those observed in the last 9 sites seem to differ. This may be due to the increased air exchange rates in the first 4 sites. In future research, measurement of the ventilation and/or air exchange rates would improve the interpretation of the effects of open windows on indoor PM_2.5_ levels. Nevertheless, to our knowledge, this study is the first study to provide the average daily dose by inhalation of indoor PM_2.5_ during pork pan frying and to evaluate quantitatively the effectiveness of ventilation in Korean residential kitchens while pan-frying meat.

## 5. Conclusions

Our study provided quantitative evidence that, in South Korea, the probability of having high ADD due to exposure to indoor PM_2.5_ during the pan-frying process is likely to be reduced by half with a forced-air stove hood at home. Ventilation through a window has a relatively minor impact on daily exposure. Operating a forced-air stove hood system is highly recommended for protecting homemakers from high PM_2.5_ exposure levels during the pan-frying process in South Korean homes.

## Figures and Tables

**Table 1:** Characteristics of sampling sites.

Site No.	House Type	Area (m^2^)	Height (m)	Indoor Smoking	Air Conditioner
1	Single house	66.0	2.4	No	No
2	Apartment	52.8	2.0	No	No
3	Apartment	52.8	2.0	No	No
4	Single house	66.0	2.0	No	No
5	Single house	66.0	2.5	No	No
6	Apartment	112.2	2.2	No	No
7	Multi units	92.4	2.2	No	No
8	Single house	108.9	2.0	No	No
9	Single house	66.0	2.5	No	No
10	Single house	52.8	2.5	No	No
11	Apartment	112.2	2.3	No	No
12	Apartment	108.9	2.3	No	No
13	Apartment	92.4	2.3	No	No

**Table 2:** Distributions of average daily doses (ADDs) from Monte-Carlo simulation using the distribution of indoor PM_2.5_ levels observed under different ventilation conditions, as well as outdoor PM_2.5_ levels obtained from urban roadsides.

Caption	ADD (mg/kg·Day)			PM_2.5_ Concentration (mg/m^3^) *		Exposure Time (min/Day)	Exposure Frequency (Weekly)	Life Time Exposure Duration ** (Year)
	from Equation (1)	from Simulation (*n* = 10,000)		from Measurements				
		Median (IQR) Mean (95% CI)		Median (IQR) Mean ± SD				
No ventilation	0.0481	0.0417 (0.0269–0.0628)	0.0496 (0.0490, 0.0502)	4.51 (2.24–5.64)	3.83 ± 1.98	65	1	35
One window open	0.0274	0.0221 (0.0138–0.0353)	0.0280 (0.0276, 0.0284)	1.82 (1.35–3.28)	2.18 ± 1.38	65	1	35
Two windows open	0.0212	0.0164 (0.0096–0.0276)	0.0217 (0.0213, 0.0221)	1.93 (0.42–2.51)	1.69 ± 1.29	65	1	35
Forced-air stove hood	0.0100	0.0070 (0.0038–0.0127)	0.0070 (0.0068, 0.0072)	0.51 (0.13–1.33)	0.79 ± 0.74	65	1	35
Urban roadside ***	0.0046	NA	NA	0.05		480	7	35

IQR: interquartile range, CI: confidence interval, SD: standard deviation, NA: not available. * The distributions of PM_2.5_ concentrations were obtained from the 13 PM_2.5_ median concentration values for 13 sampling sites (real-time based 2 h measuring with pan-frying for the first 9 min), Median (IQR) background PM_2.5_ concentration were 0.022 (0.012–0.042) mg/m^3^. ** For housewife aged 25 years; *** Data from Air Korea (2016) [22].

**Table 3:** Distributions of indoor PM_2.5_ concentrations during the first 9 min of cooking period and the dissipation kinetics after cooking was completed.

Type of Ventilation	PM_2.5_ Concentration (μg/m^3^) during the First 9 min Fan-Prying Period			Dissipation Kinetics ((μg/m^3^)/min) after Cooking Was Completed		
	Median	25%ile	75%ile	Median	25%ile	75%ile
No ventilation	5142.2	2958.2	9228.7	38.2	26.6	79.4
One window open	4970.6	1668.6	6990.8	47.4	17.0	84.6
Two windows open	3777.2	1348.8	6192.6	54.7	20.1	99.9
Forced-air stove hood	1159.6	183.3	2269.2	55.2	6.5	78.7

## References

[1] Statistics Korea The Cause of Death Statistics 2013. http://kostat.go.kr/portal/korea/kor_nw/2/1/index.board?bmode=read&aSeq=330181.

[2] Yu I.T.S., Chiu Y.L., Au J.S.K., Wong T.W., Tang J.L. (2006). Dose-response relationship between cooking fumes exposures and lung cancer among Chinese nonsmoking women. Cancer Res..

[3] Abt E., Suh H.H., Allen G., Petros K. (2000). Characterization of Indoor Particle Sources: A Study Conducted in the Metropolitan Boston Area. Environ. Health Perspect..

[4] Long C.M., Suh H.H., Koutrakis P. (2000). Characterization of Indoor Particle Sources Using Continuous Mass and Size Monitors. J. Air Waste Manag. Assoc..

[5] Lee J.B., Kim H.J., Jung K., Kim S.D. (2009). Emission Characteristics of Particulate Matters from Under-fired Charbroiling Cooking Process using the Hood Method. J. Environ. Health Sci..

[6] Rim D., Wallace L.A., Nabinger S., Persily A. (2012). Reduction of exposure to ultrafine particles by kitchen exhaust hoods: The effects of varying flow rates, particle size, and burner position. Sci. Total Environ..

[7] Jarvis D., Chinn S., Luczynska C., Burney P. (1996). Association of respiratory symptoms and lung function in young adults with use of domestic gas appliances. Lancet.

[8] Travers M.J. Casino Air Monitoring Study East Saint Louis, Illinois. htp://tobaccofreeair.org/documents/IllinoisCasinoAirMonitoringReport.pdf.

[9] IARC HIGH-TEMPERATURE FRYING, Leon, France. http://monographs.iarc.fr/ENG/Monographs/vol95/mono95-7.pdf.

[10] Ministry of Environment Source Appointment of PM_2.5_. National Institute of Environmental Research. Sejong, South Korea. http://www.prism.go.kr/homepage/researchCommon/retrieveResearchDetailPopup.do;jsessionid=B45E976CBC454200C4532494792FB6EB.node02?research_id=1480000-200900330.

[11] National Institute of Animal Science Livestock Management Issue Report. http://www.nias.go.kr/front/prboardView.nias?cmCode=M090814150936066&boardSeqNum=278&columnName=&searchStr=&currPage=1.

[12] Taner S., Pekey B., Pekey H. (2013). Fine particulate matter in the indoor air of barbeque restaurants: Elemental compositions, sources and health risks. Sci. Total Environ..

[13] Kim D.Y. Levels Air Pollutants at Barbeque Restaurants. http://www.gri.re.kr/korea/jsp/policy/gri_view.jsp?idx=2917:3529&go=13&gogroup=.

[14] Korea Gas Corporation. http://www.kogas.or.kr.

[15] Pakey B., Bozkurt Z.B., Pekey H., Dogan G., Zararsiz A., Efe N., Tuncel G. (2010). Indoor/outdoor concentrations and elemental composition of PM_10_/PM_2.5_ in urban/industrial areas of Kocaeli city, Turkey. Indoor Air.

[16] Kim S., Sohn J., Lee K. (2012). Exposure to Particulate matters (PM_2.5_) and airborne nicotine in computer game rooms after implementation of smoke-free legislation in South Korea. Nicotine Tob. Res..

[17] Dacunto P.J., Cheng K.C., Acevedo-Bolton V., Jiang R.T., Klepeis N.E., Repace J.L., Ott W.R., Hildemann L.M. (2013). Real-time particle monitor calibration factors and PM_2.5_ emission factors for multiple indoor sources. Environ. Sci. Process. Impacts.

[18] United States Environmental Protection Agency Guidelines for Exposure Assessment. http://cfpub.epa.gov/ncea/cfm/recordisplay.cfm?deid=15263#Download.

[19] Jang J.Y., Jo S.N., Kim S.Y., Kim S.J., Cheong H.K. Korean Exposure Factors Handbook. http://m.riss.kr/search/detail/DetailView.do?p_mat_type=d7345961987b50bf&control_no=1f310160f0f75c15ffe0bdc3ef48d419.

[20] Kim S.R., Halden R.U., Buckley T.J. (2007). Volatile Organic Compounds in Human Milk: Methods and Measurements. Environ. Sci. Technol..

[21] Statistics Korea Average Time Spent on Activities by Age Group—20 Years Old & Over, 65 Years Old & Over (Cont’d). http://kostat.go.kr.

[22] Ministry of Environment Korea National Ambient Air Quality Standard. http://www.me.go.kr/mamo/web/index.do?menuId=586.

[23] Lee S.C., Li W.M., Chan L.Y. (2001). Indoor air quality at restaurants with different styles of cooking in metropolitan Hong Kong. Sci. Total Environ..

[24] Hu W., Downward G.S., Reiss B., Xu J., Bassig B.A., Hosgood H.D., Zhang L., Seow W.J., Wu G., Chapman R.S. (2015). Personal and Indoor PM_2.5_ Exposure from Burning Solid Fuels in Vented and Unvented Stoves in a Rural Region of China with a High Incidence of Lung Cancer. Environ. Sci. Technol..

[25] Kim B.K., Yun D.M., Kim S.R. (2014). Assessment of Secondhand Smoke Exposure Levels by Measuring PM_2.5_ Concentration at Various Smoking Hotspot Place Inside and Outside Campus. J. Korean Soc. Res. Nicotine Tob..

[26] Statistics Korea. http://kostat.go.kr/portal/korea/index.action.

